# Retrospective analysis of clinical outcome of 100 inoperable oral cavity carcinoma treated with definitive concurrent chemoradiotherapy with or without induction chemotherapy

**DOI:** 10.3332/ecancer.2023.1630

**Published:** 2023-11-16

**Authors:** Vachaspati Kumar Mishra, Ajeet Kumar Gandhi, Madhup Rastogi, Rakhi Verma, Rohini Khurana, Rahat Hadi, Vikas Sharma, Akash Agarwal, Anoop Kumar Srivastava

**Affiliations:** 1 Department of Radiation Oncology, Dr Ram Manohar Lohia Institute of Medical Sciences, Lucknow 226010, Uttar Pradesh, India; 2 Department of Surgical Oncology, Dr Ram Manohar Lohia Institute of Medical Sciences, Lucknow 226010, Uttar Pradesh, India

**Keywords:** definitive concurrent chemoradiotherapy, carcinoma oral cavity, inoperable

## Abstract

**Objectives:**

The management of inoperable oral cavity squamous cell carcinoma (OC-SCC) is onerous. We aimed to retrospectively analyse the outcome of our cohort of inoperable OC-SCC treated with definitive concurrent chemoradiotherapy (CTRT) with or without induction chemotherapy (IC).

**Methods:**

Data of 100 patients (January 2017 to May 2022) of histopathologically proven inoperable OC-SCC treated with definitive CTRT with weekly cisplatin 40 mg/m^2^ were retrieved from our departmental archives. Radiotherapy (RT) was delivered with three-dimensional conformal plan (66–70 Gy). Toxicities were evaluated using acute morbidity scoring criteria of Radiation Therapy Oncology Group. The response was evaluated as per WHO criteria. Progression free survival (PFS) was calculated from the date of the start of treatment (IC/CTRT) using Kaplan Meier method.

**Results:**

Median age was 45 years (range 30–80 years). The primary site was oral tongue (59%), retro-molar trigon (15%), buccal mucosa (15%) and others (11%). The stage was III: IVA: IVB in 16:70:14 patients respectively. 72% patients received IC (platinum ± 5 FU ± taxane). Grade 3 skin toxicity, oral mucositis and dysphagia was noted in 13 (13%), 19 (19%) and 13 (13%) patients respectively. The median follow-up duration was 30.5 months (range 6–62 months). Complete response (CR), partial response, progressive disease and death at the time of the last follow-up were 49%, 25%, 15% and 11% respectively. 2-year PFS rate was 49.5%. Stage III patients had a higher CR rate (81.2% versus 42.8%; *p* = 0.0051) and higher 2-year PFS (81.2% versus 46.4%; *p* = 0.0056) in comparison to stage IV patients.

**Conclusion:**

Inoperable patients of OC-SCC treated with definitive CTRT with or without IC yielded CR in approximately half of patients with acceptable toxicity profiles.

## Introduction

Carcinoma of the lip and oral cavity is the second most frequent cancer in India and the third leading cause of cancer-related deaths. With an annual incidence of 135,929 cases, this cancer is becoming a major health concern in our country [1]. The most prevalent histological type is oral cavity squamous cell carcinoma (OC-SCC) [2]. Tobacco chewing, smoking and alcohol intake are significant risk factors for the development of OC-SCC [3, 4].

The current standard of care for locally advanced OC-SCC (stage III–IVA) is surgery with adjuvant radiotherapy (RT) with or without concurrent chemotherapy [5, 6]. However, the management of locally advanced inoperable OC-SCC (technically unresectable, medically inoperable or due to patient preference) is not standardised. These patients are generally managed with varied treatment options ranging from definitive RT with or without chemotherapy, palliative RT or systemic therapy with either alone or a combination of chemotherapy, immunotherapy and targeted therapy. Selected inoperable OC-SCC patients may be treated with definitive concurrent chemoradiotherapy (CTRT) with or without induction chemotherapy (IC) depending on the performance status of the patient and tumour extent. The treatment outcome of CTRT has been inconclusive, as several studies have reported a wide range of 5-year overall survival (OS) rates, ranging from 29% to 76% [7–10]. Treating these patients has proven to be exceptionally challenging, with lower survival rates compared to other head and neck cancers [11, 12]. These patients may require a higher curative dose of radiation, but the delivery of high radiation dose is often limited by critical organs at risk in the vicinity of the oral cavity like parotid glands, mandible and dysphagia aspiration-related structures; higher doses to these organs may lead to significant morbidities like xerostomia, osteoradionecrosis, acute and late dysphagia [13–15].

Definitive CTRT with or without IC has been widely used as a management option in other subsites of head and neck cancers, and results have shown acceptable clinical outcome along with descent quality of life with this modality [16, 17]. Employing definitive CTRT in OC-SCC has been met with concerns regarding its clinical outcome and potential adverse effects [11, 18–21] and hence there is a paucity of robust evidence evaluating this approach.

Therefore, we intended to retrospectively evaluate the clinical outcome in terms of complete response (CR) rates with progression-free survival (PFS) and toxicity profiles of our cohort of inoperable OC-SCC treated with definitive CTRT with or without IC at a tertiary cancer institute in India.

## Materials and methods

### Patients

Data of 186 inoperable OC-SCC patients were retrieved from our departmental archives. 118/186 patients received IC, of which 46 patients (38.9%) underwent surgery after IC (excluded from analysis). 40/186 (21.5%) patients received palliative RT and or systemic therapy (excluded from analysis). Finally, 100 patients (28 receiving upfront CTRT and 72 receiving IC followed by CTRT) formed the study cohort for our present retrospective analysis (January 2017 to May 2022).

Patients had locally advanced disease (stage III–IV disease), with a Karnofsky performance status (KPS) ≥70, haemoglobin ≥10 g/dL, total leukocyte count ≥4,000/mm, platelet count ≥100,000/mm^3^, creatinine clearance ≥60 mL/minute and normal liver function test. Patients who were either inoperable after IC or those who received upfront CTRT were included in this study. Patients deemed resectable after IC, non-squamous histology, those with distant metastases, those receiving palliative RT, with synchronous/metachronous malignancy and prior history of head and neck radiation were excluded.

Inoperable OC-SCC patients are routinely discussed in the multidisciplinary tumour board at our institute. An attempt is always made to offer surgical treatment to these patients. In general, we offer IC to those with borderline operable disease (those with mid or low infratemporal fossa involvement, minimal masticator space involvement, minimal involvement of the base of tongue or adjacent structures) or those who are presumed to be operable after down staging of tumour with IC. Based on patient factors like age, performance status, comorbid conditions and tumour factor like extent and stage of disease along with a discussion with patients about potential benefit/adverse event, we decide to offer these patients IC or definitive CTRT. The decision of chemotherapy regimen depends on the performance status of the patient. In general, response assessment was done with clinical examination after each cycle and radiological assessment was done after 2–3 cycles.

In patients receiving IC, response assessment with clinical and radiological imaging is done after 2–3 cycles. Patients deemed unfit for surgery either due to unresectable primary/nodal disease (high or mid infratemporal fossa involvement, masticator space involvement either due to temporalis or lateral pterygoid muscle involvement, the disease having skull base invasion, internal carotid artery encasement, pterygoid plate involvement, the base of tongue or root of tongue involvement, fixed neck node with extracapsular extension) or medical inoperability owing to patient factors but deemed suitable to receive full course of definitive CTRT were given definitive CTRT.

Pre-treatment evaluation was done with detailed history and complete physical as well as radiographic assessment of the primary tumour, neck and chest. Patients were routinely referred for pre-treatment dental evaluation. All patients were staged as per the seventh edition of the American Joint Committee on Cancer [22].

### Treatment

IC was administered with 2–3 cycles of (platinum ± 5 FU ± taxane) repeated every 3 weeks. Definitive CTRT was given in the form of external beam RT (EBRT) or brachytherapy (BT) boost with EBRT along with weekly cisplatin. EBRT was delivered using 6-MV photons to a dose of 66–70 Gy, 2-Gy per fraction, 5 days per week by using a linear accelerator (Infinity and Synergy; Elekta, Crawley, UK) with a multi-leaf collimator width of 1 cm at the isocentre. Patients were treated with three-dimensional conformal RT (3-D CRT) using bilateral parallel opposed fields in two phases, in which 44 Gy was delivered in the first phase and a coned down boost of 22–26 Gy was delivered in the second phase with or without supplemental posterior neck electron boost. Concurrent chemotherapy was given with weekly intravenous cisplatin at 40 mg/m^2^. The dose was modified according to weekly assessment of creatinine clearance prior to each applied dose.

Those patients who were medically inoperable or preferred non-surgical treatment with T2N0-1 oral tongue/buccal mucosa primary (tumour size of less than <4 cm and located ≥5 mm away from mandible) were offered BT boost along with CTRT. BT was delivered in the form of interstitial BT. In the cohort of EBRT plus BT boost patients, 45–50 Gy was delivered by EBRT followed by BT boost with a dose of 16–20 Gy in 4–5 fractions.

All patients were on a regular follow-up. Follow-up was according to our institutional protocol, 3 monthly for the first year, every 4 months for the second year, every 6 months for the third to fifth years, and then annually, thereafter. History and clinical examination were done at all these followups. All patients underwent a contrast-enhanced computed tomography of the face and neck region at 3 months after completion of RT or earlier on clinical suspicion of progression of disease. Routine imaging was not done thereafter during followup and was advised based on clinical suspicion of disease recurrence or progression.

### Statistics

The primary end point was to evaluate the treatment efficacy in terms of response to the treatment and secondary end point were assessment of PFS, compliance of treatment, acute and late toxicities. Clinical response was evaluated as per WHO criteria [23]. PFS was defined as the time from the day of the start of treatment (IC/CTRT) to the date of progression of disease or death due to any cause. All survival analyses were performed by using Kaplan–Meier method. Log-rank test was used to study the impact of prognostic variables on response rate and PFS. Cox regression analysis was used for multivariate analysis. *p*-value < 0.05 was considered statistically significant. Compliance with treatment was defined as receipt of ≥66 Gy of RT and ≥5 cycles of concurrent chemotherapy. Acute toxicities and late toxicities were evaluated according to the Radiation Therapy Oncology Group criteria. Toxicity analyses were presented as crude rates and percentages. Statistical analysis was done by Statistical Package for Social Sciences software (Version 23.0; IBM).

## Results

### Patient and treatment characteristics

Patient characteristics are summarised in Table 1. 72 patients (72%) received IC (platinum ± 5 FU ± taxane) q 3 weekly, median number of cycles was 3 (range 2–4). All patients received concurrent chemotherapy with a median number of 5 cycles (range 2–7). Median RT dose was 70 Gy (range 66–70 Gy). Five patients (three oral tongue and two buccal mucosa) received RT in the form of EBRT and BT boost. The compliance rate of the prescribed treatment was 70%. All patients received ≥66 Gy RT dose. Concurrent chemotherapy cycles received ≥5:4:3:2 was 70:20:6:4 patients respectively.

### Clinical outcome

**Figure 1.**
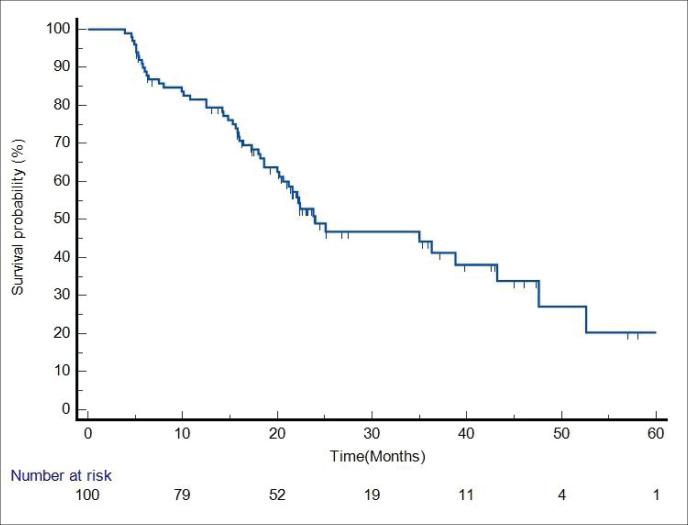
PFS of entire cohort.

**Figure 2.**
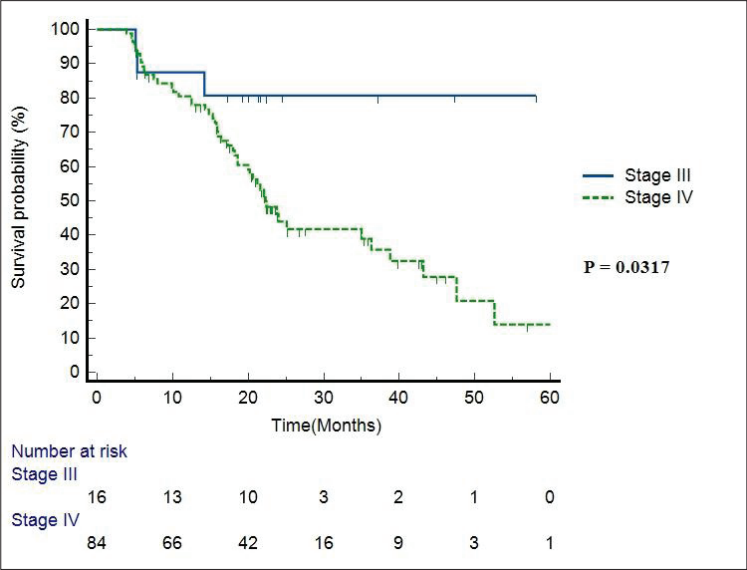
PFS in stage III versus stage IV patients.

Median follow-up duration was 30.5 months (range 6–62 months). Table 2 shows the patient response rate to the treatment. CR, partial response (PR), progressive disease (PD) and death at the time of last follow-up were 49%, 25%, 15% and 11%, respectively. All five patients receiving EBRT plus BT boost achieved CR and were disease free at the time of last follow up. PFS of entire cohort is represented in Figure 1. Median PFS was 24.2 months (95% confidence interval (CI) 18–49.4 months). 2-year PFS rate was 49.5%. Table 3 shows the prognostic factors related with CR and PFS on univariate analysis. Both T4 and stage IV disease were associated with a statistically significant lower CR rate and 2-year PFS as compared to stage III disease. Figure 2 shows the variation of PFS in stage III versus stage IV patients. On multivariate analysis of T2/3 versus T4 disease and N0/1 versus N2/N3 had significantly better PFS (hazard ratio (HR) = 3.4; 95% CI, 1.2–9.4; *p* = 0.01) and (HR = 2.1; 95% CI, (1.1–3.9); *p* = 0.019), respectively.

### Toxicity analysis

Grade ≥ 3 acute skin toxicity, oral mucositis and dysphagia was noted in 13 (13%), 19 (19%) and 13 (13%) patients respectively. No treatment-related deaths were noted. Xerostomia grade ≥ 2, grade 1 and grade 0 were seen in 27 (27%), 56 (56%) and 17 (17%) of patients respectively, and subcutaneous fibrosis grade ≥ 2, grade 1 and grade 0 were seen in 18 (18%), 52 (52%) and 30 (30%) of patients respectively. Late dysphagia grade ≥ 2 was noted in 16 (16%) patients.

## Discussion

This retrospective study aimed to analyse the clinical outcome of inoperable OC-SCC treated with definitive CTRT with or without IC in a tertiary cancer centre in India. Data from the Indian Council for Medical Research have highlighted that approximately 70% of patients with OC-SCC in India present with locally advanced stage and majority of them are treated with palliative intent [24]. In our study, 84% of patients had stage IV disease and most common primary site was oral tongue (59%) followed by retro-molar trigone (RMT) (15%) and buccal mucosa (15%).

Selected inoperable OC-SCC patients may be treated with IC followed by surgery or definitive CTRT. In the study by Patil *et al* [25], an attempt had been made to downstage the malignancy with IC so that surgical resections with negative margins could be achieved and they have reported that IC was effective in converting technically unresectable oral cavity cancer to operable disease in approximately 40% of patients and was associated with significantly improved OS in comparison to nonsurgical treatment. In our study, 38.9% (46 of 118 patients receiving IC) patients underwent surgery after IC. IC may still be used for technically/borderline resectable patients to increase their chances of undergoing surgery with potentially better outcome.

We did not observe a statistical difference in the clinical outcome of patients treated with definitive CTRT or IC followed by definitive CTRT in terms of response rates or PFS. It is important to note that these IC treated patients were selected patient cohort (who did not receive significant response from IC and lying between those suitable for surgery and palliative intent of therapy) and may not directly represent the clinical outcome of patients treated with IC followed by definitive CTRT. However, patients with inoperability due to medical co-morbidities or due to patient preference may be taken up for upfront CTRT rather than IC followed by CTRT, thus sparing them of additional cost and toxicity.

Some retrospective analyses have investigated the efficacy of definitive CTRT for OC-SCC [7, 10, 11, 19–21]. Cohen *et al* [10] reported 5-year PFS of 51%, and OS of 56%. A study by Scher *et al* [18] observed a 5-year OS rate of 15% and 5-year loco-regional control (LRC) of 37%. Another study by Foster *et al* [11] showed 5-year LRC of 78.6%, PFS of 51.7% and OS of 63.2%. However, these studies did not report the CR rate, which is an important parameter for treatment efficacy. Our study reported CR rate of 49% and 2-year PFS rate of 49.5% with definitive CTRT. The results of our study have been summarised along with the results of contemporary series in Table 4. Clinical outcome of our analysis is comparable with those of other institutions.

Few studies have reported the association of higher T-stage, younger age, positive nodal status and RT doses (<70 Gy) to be poor prognostic factors for PFS [26–28] but results of our study found that only T- stage and nodal status have significant effect on PFS in multivariate analysis. The CR rates in patients with T4b and N3 disease were less than 15% and hence it may be futile to treat these patients with a protracted course of IC followed by CTRT. These patients have a bad biology of disease and should be treated with palliative intent with either RT/chemotherapy. We could not find a statistically significant effect of age and IC on treatment outcomes in our cohort of patients. That could be attributed possibly to heterogeneity in the study population.

The incidence of acute and late toxicities in our study is consistent with results of other studies [11, 27, 28]. Oral mucositis was the most observed >grade 2 acute toxicity (19%) as majority of patients in our study had oral tongue as primary site and patients received treatment by 3-D CRT technique which could be responsible for higher reactions (oral mucositis and dysphagia). Late toxicities are often ignored and under-reported which tend to get worse over a period of time, in our study ≥2 grade xerostomia, subcutaneous fibrosis and late-dysphagia was noted in 27%, 18% and 16% of patients respectively which are comparable with other studies [11, 28].

Based on our results, we suggest that definitive CTRT in locally advanced OC-SCC is a feasible approach as the compliance rate of our study was 70%. Certain limitations associated with our analysis have been mentioned. The retrospective nature of the analysis may have led to a selection bias of the patients and underreporting of certain treatment parameters like late toxicities. Additionally, our report lacks data on comprehensive assessment of OS which is a crucial endpoint for evaluating the clinical efficacy of any study. The unavailability of OS data for majority of patients hindered us from reporting it adequately. Patients of inoperable OC-SCC have limited survival after progression on definitive CTRT and PFS may be presumed to be a surrogate of OS in these cohort of patients. These limitations reinforce the significance of reporting single institutional data, which can eventually be consolidated through meta-analysis. Our study's strengths lie in the uniformity of an underrepresented cohort of OC-SCC treated with a protocolised management guideline. We also reported CR rates which is a relatively underexplored aspect in clinical research of inoperable OC-SCC. This study will further enhance the perspicacity of the clinical outcome and toxicity profiles of patients treated with definitive CTRT in locally advanced OC-SCC.

The management of patients with head and neck squamous cell carcinoma including OC-SCC is greatly influenced by factors such as tumour biology, the micro-environment, stage and other prognostic indicators. Numerous studies have explored potential biomarkers like PDL-1, EGFR etc., in this context [29–33]. In recent years, there have been an extensive investigation aimed at identifying prognostic and predictive biomarkers. However, it's important to note that personalised treatments for OC-SCC remain limited. While few targeted therapies, such as anti-EGFR and anti-PDL-1 have been approved, there is a paucity of data for the same in inoperable OC-SCC. Future research may focus on combination of RT concurrent with immunotherapy [31], or concurrent with novel anti-EGFR inhibitors like nimotuzumab [32].

## Conclusion

The findings of our study suggest that inoperable patients of OC-SCC treated with definitive CTRT with or without IC yields CR in approximately half of the patients with acceptable toxicity profiles. Future research including studies on biomarkers for better patient selection and treatment personalisation is warranted.

## Conflicts of interest

The authors have no conflicts of interest to declare.

## Funding

None.

## Author contributions

Conceptualisation: AKG, MR, VKM; investigation and methodology: AKG, MR, VKM, RK, RH, RV, AKS; supervision: AKG, MR, VKM, RK, RH, RV, AKS; data curation: VKM, AKG, MR, RK, RH; analysis and interpretation: AKG, MR, VKM; writing of the original manuscript: VKM, AKG; writing of the review and editing: AKG, VKM, MR. All authors have proofread and approved the final version.

## Figures and Tables

**Table 1.** Patient characteristics.

Patient characteristics	Distribution (*n* = 100)
Median age (range)	45 years (range 30–80 years)
Male: female	87:13
Median KPS (range)	90 (range 80–90)
Differentiation (well: moderate: poor)	38 (38%):51 (51%):11 (11%)
Primary site Oral tongue: RMT: buccal mucosa: others	59 (59%):15 (15%):15 (15%):11 (11%)
T size – T2:T3:T4a:T4b N status – N0:N1:N2:N3 Stage – III:IVA:IVB	7 (7%):25 (25%):61 (61%):7 (7%) 38 (38%):22 (22%):33 (33%):7 (7%) 16 (16%):70 (70%):14 (14%)
IC regimen (*n* = 72) TPF:TP:PF	35 (48.6%):27 (37.5%):10 (13.9%)

KPS = Karnofsky performance status; RMT = retro-molar trigone; TPF = taxane + platinum + 5-FU; TP = taxane +platinum; PF = platinum + 5-FU; TF = taxane + 5-FU

Staging has been done as per AJCC 7th edition

**Table 2.** Response rates as per the T, N and overall stage of the patients.

	CR	PR	PD	Death
T2 (*n* = 7)	5 (71.4%)	1 (14.3%)	0 (0%)	1 (14.3%)
T3 (*n* = 25)	20 (80%)	2 (8%)	1 (4%)	2 (8%)
T4a (*n* = 61)	23 (37.8%)	21 (34.4%)	11 (18%)	6 (9.8%)
T4b (*n* = 7)	1 (14.3%)	1 (14.3%)	3 (42.8%)	2 (28.6%)
N0 (*n* = 38)	21 (55.3%)	10 (26.3%)	5 (13.1%)	2 (5.3%)
N1 (*n* = 22)	12 (54.4%)	4 (18.1%)	3 (13.6%)	3 (13.6%)
N2 (*n* = 33)	15 (45.5%)	8 (24.2%)	6 (18.2%)	4 (12.1%)
N3 (*n* = 7)	1 (14.3%)	3 (42.8%)	1 (14.3%)	2 (28.6%)
Stage-III (*n* = 16)	13 (81.3%)	1 (6.2%)	0 (0%)	2 (12.5%)
Stage IVa (*n* = 70)	34 (48.5%)	20 (28.5%)	11 (15.7%)	5 (7.1%)
Stage IVb (*n* = 14)	2 (14.2%)	4 (28.6%)	4 (28.6%)	4 (28.6%)

CR = Complete response; PR = partial response; PD = progressive disease

**Table 3.** Univariate analysis of the impact of prognostic variables on CR rates and 2-year PFS.

Variables	CR rates at last date of follow-up		2-year PFS	
	CR (%)	*p*-value	PFS	*p*-value
T-size: T2–T3 versus T4	78.1% versus 35.3%	0.0008	80.2% versus 41.6%	0.0009
Node: N0–N1 versus N2–N3	55% versus 42.1%	0.1018	56.1% versus 39.5%	0.0478
Stage: III versus IV	81.2% versus 42.8%	0.0051	81.2% versus 46.4%	0.0056
Age: ≤50 versus >50 years	52.6% versus 53%	0.5734	46.9% versus 52.9%	0.2563
IC: Yes versus no	57.1% versus 52.8%	0.3122	45.8% versus 42.8%	0.3932
RT dose: ≤66 versus >66 Gy	48.14% versus 50%	0.8542	50% versus 51.8%	0.4287
Concurrent chemotherapy cycles: <5 versus ≥5 cycles	52.7% versus 41.37	0.3324	52.11% versus 63.15%	0.2314

IC = Induction chemotherapy; RT = radiotherapy; CR = complete response; PFS = progression free survival

**Table 4.** Studies depicting the clinical outcome of patients treated with definitive CTRT.

Studies	Number of patients	Type of study	Median follow up	Clinical outcome
Scher *et al* [18]	73	Retrospective	73.1 months	5-year OS – 15% 5-year LRC – 37%
Spiotto *et al* [20]	2,091	Retrospective	17.3 months	3-year OS – 37.8%
Tangthongkum *et al* [21]	61	Retrospective	-	5-year OS – 24%
Cohen *et al* [10]	39	Retrospective	83 months	5-year PFS – 51% 5-year OS – 56%
Foster *et al* [11]	140	Retrospective	5.7 years	5-year OS – 63.2% 5-year PFS – 58.7% 5-year LRC – 78.6%
Present study	100	Retrospective	30.5 months	2-year PFS – 49.5% 2-year CR rate – 50%

OS = Overall survival; LRC = loco-regional control; PFS = progression free survival; CR = Complete response rates
